# Recalibrating vascular malformations and mechanotransduction by pharmacological intervention

**DOI:** 10.1172/JCI160227

**Published:** 2022-04-15

**Authors:** Salim Abdelilah-Seyfried, M. Luisa Iruela-Arispe, Josef M. Penninger, Elisabeth Tournier-Lasserve, Miikka Vikkula, Ondine Cleaver

**Affiliations:** 1 Institute of Biochemistry and Biology, Potsdam University, Potsdam, Germany.; 2 Department of Cell and Developmental Biology, Northwestern University Feinberg School of Medicine, Chicago, Illinois, USA.; 3 Department of Medical Genetics, Life Sciences Institute, University of British Columbia, Vancouver, British Columbia, Canada.; 4 INSERM UMR 1141 Neurodiderot, University of Paris, Paris, France.; 5 AP-HP, Department of Genetics of Neurovascular Diseases, Hôpital Saint-Louis, Paris, France.; 6 Human Molecular Genetics, de Duve Institute, Brussels, Belgium.; 7 Department of Molecular Biology, UT Southwestern Medical Center, Dallas, Texas, USA.

## Introduction

Circulation of blood throughout the cardiovascular system results in biomechanical forces that profoundly influence vessel development and maintenance. Fluid shear stress along the inner lining of blood vessels imparts mechanical forces upon vascular cells. Endothelial cells (ECs), which line all blood and lymphatic vessels, are exquisitely equipped to sense mechanical forces and to transduce these stimuli into biochemical signals, which control their proliferation, migration, cytoskeleton organization, and cell-cell adhesion. Vascular malformations can arise when patterns of blood flow change or when vascular cells develop a disturbed response to hemodynamic forces due to genetic mutations. For instance, genetic evidence suggests that mutations causing dysregulated RAS/MAPK and nitric oxide (NO) signaling within vascular cells lead to malformations and dysplasias. The influence of biomechanical cues on genetically vulnerable cells provides a promising therapeutic avenue. Vascular malformations are often incurable, and patients experience lifelong chronic pain, disfigurement, or even premature death. A major obstacle to developing useful therapies is our poor understanding of the molecular underpinnings of these vascular defects. Tangible therapeutic approaches may be based on the novel idea that vascular cell responses to blood flow can be normalized using pharmacological modulation.

## Vascular malformations and dysplasias arise when mechanotransduction goes awry in endothelial cells

Brain vascular malformations/dysplasias (VaMs) can lead to stroke with severe consequences or sudden death at any age (1, 2). In other organs, VaMs can also cause hemorrhages, deformities, and severe chronic pain. A major impediment to the development of treatments is the poor understanding of the molecular pathways and factors, including mechanical forces, that stimulate their development and growth. Vascular cells are sensitive to the deleterious combination of germline mutations present from conception (hereditary VaMs) and somatic mutations that arise later in life (sporadic VaMs). Perplexingly, mutated vascular cells sometimes remain dormant despite the presence of causative mutations. Mounting evidence shows that within a defined deleterious genetic context, biomechanical forces due to fluid shear stress catalyze the formation of VaMs.

Blood flow creates two main hemodynamic forces that impact vascular cells and shape blood vessel architecture: frictional forces along the longitudinal axis of the inner vessel surface (referred to as fluid shear stress) and blood pressure that stretches the vessel wall across its diameter. Cells lining major arterial blood vessels close to the heart experience pulsatile flow at high speed and under high pressure, while venous and capillary ECs are exposed to much lower pressure flow, with almost no pulsatility. While flow within the large-caliber vessels is largely laminar (parallel to the vessel), oscillatory and disturbed flow occurs at vascular branching points or at valvular structures. Under both types of mechanical stress, ECs experience stretch and pulling at cell-cell junctions, which are themselves anchored to the cytoskeleton. Multiple sensors exist both along the plasma membrane and at cell junctions that relay physical forces across the cell cytoplasm to the nucleus. It is noteworthy that the endothelium, which has been shown over the last few decades to be highly regionalized and heterogeneous (3), is able to withstand and thrive under these varied conditions.

Physical forces caused by blood flow are sensed and transduced by ECs and smooth muscle cells via multiple intracellular signaling transduction pathways (4). These signaling processes engage a wide range of molecular events — including modifications of adaptor proteins at adherens junctions or focal adhesions, local changes in actomyosin network contractility, or induction of biomechanical signaling that regulates downstream pathways such as NO and cGMP signaling. NO and cGMP are key determinants of cellular contractility/stiffness, vessel caliber, and blood pressure regulation (5, 6). Although vascular ECs likely harbor the same basic machinery across the vascular tree, it is clear that different vessel beds interpret and respond to blood flow in different ways, depending on their location and cellular context.

The last decade has seen mounting evidence linking blood flow patterns and cardiovascular malformations. For instance, patients with constant-flow left ventricular assist devices that produce a nonpulsatile blood flow exhibit gastrointestinal arteriovenous malformations (AVMs) and stroke more frequently than patients using pulsatile flow devices (7, 8). Further evidence for the role of mechanobiology in several inherited vasculopathies has emerged with the discovery that pathogenic changes to RAS/MAPK pathway and NO signaling cause disturbed biomechanical signaling. RAS/MAPK signaling and NO signaling (i) have surfaced as central pathways that integrate biomechanical stimuli with cell cycle regulation, cell fate responses, and migration (9–11); (ii) are defective in many types of VaMs (12, 13); and (iii) are the targets of well-characterized drugs, making them excellent tools for manipulation. For instance, cerebral cavernous malformations (CCMs) are caused by inherited mutations in the *CCM1–3* genes (14, 15), resulting in activation of MAPK and *KLF2/4* signaling (16, 17). The latter are major blood flow–responsive genes. Mutations in MAPK and NO pathway genes were also reported in moyamoya angiopathy (MMA), a cerebrovascular condition affecting internal carotid artery bifurcations (13, 18, 19). *MAP2K1* and *NOTCH* mutations cause peripheral AVMs (20, 21), while somatic *KRAS* mutations elicit cerebral AVMs (22). NOTCH is an arterial EC mechanosensor of laminar shear stress (23). Capillary malformation AVMs (CM-AVMs) result from loss-of-function mutations in *RASA1* (24), a RAS suppressor, or its associated receptor EPHB4 (25). The identification of defective signaling pathway components in these numerous vascular malformations underscores their centrality to normal vascular homeostasis and health.

**Figure 1:**
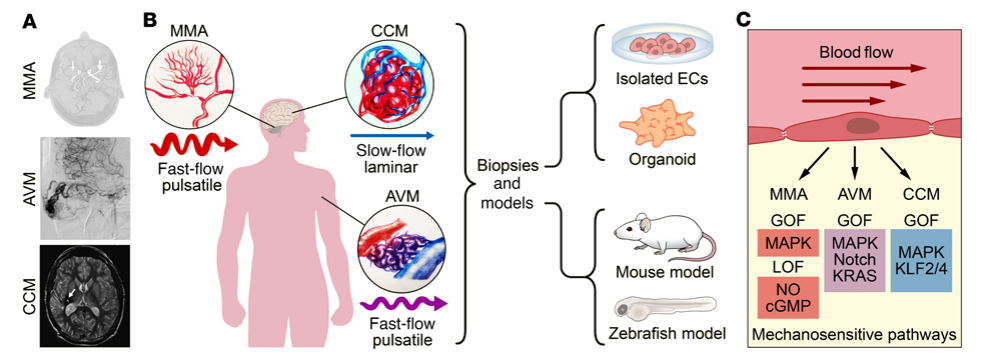
Approaches to investigating molecular mechanisms underlying vascular malformations. (**A**) Imaging of typical vascular lesions in moyamoya angiopathy (MMA), arteriovenous malformations (AVM), and cerebral cavernous malformations (CCM). (**B**) Vascular anomalies develop in different vascular beds that are subject to different hemodynamic environments. MMA lesions appear in intracranial carotid artery bifurcations; AVMs can appear anywhere in the body, including the central nervous system, in internal organs, and subcutaneously; CCMs primarily develop in the brain but can also occur in the spinal cord. Multiple parallel experimental approaches, both in vivo and in vitro, are needed to study the influence of mechanosensitive pathways on vascular malformations. (**C**) We propose that future studies are needed to interrogate the signaling pathways linked to vascular malformations, including, in vivo and in vitro, gain-of-function mutations (GOF) in the MAPK pathway (mutations observed in MMA, AVMs, and CCMs) and the Notch, KRAS (AVMs), and KLF2/4 (CCMs) pathways, as well as loss-of-function mutations (LOF) in the NO and cGMP pathways (MMA). Figure illustrated by Jose Cabrera (UT Southwestern) and Jeanne Mora Garcia (Potsdam University).

Understanding how the RAS/MAPK pathway is dysregulated in these vasculopathies should also clarify why ECs behave abnormally in response to blood flow. Specifically, CCMs occur in slow-flow cerebral venous capillaries, while AVMs and MMA are fast-flow vasculopathies; yet all of these pathologies are associated with dysregulation of MAPK signaling (Figure 1). Another critical discovery was that blood flow has a vasoprotective effect in CCM. While CCM-deficient aortic ECs of zebrafish undergo pathological growth in the absence of blood flow, this was prevented when blood flow was restored (26). Similar observations were made in a cell culture model of CCM (27). These findings demonstrate that the dysregulation of the RAS/MAPK pathway and NO signaling has different molecular and cellular consequences when vascular cells are exposed to different flow conditions. While some regions of the vasculature develop pathologies, others are protected.

Both changes in flow and manipulation of the signaling pathways involved can restore vascular function and integrity of defective vessels. In a recent study, disturbed mechanotransduction in CCM-deficient endothelium was normalized using ERK5 and MEKK3 inhibitors, which suppressed CCM lesion formation in mouse models (17). This approach demonstrated the importance of elucidating exactly how modulating mechanotransduction cascades can impact slow-flow RAS/MAPK vascular pathologies. However, we will need to develop different approaches for rewiring mechanotransduction pathways when treating fast-flow vascular diseases such as MMA, which is linked to a loss of NO signaling. Observations that the effects of biomechanical inputs can be bypassed by targeting specific pathways underscore the potential for novel small molecule–based therapeutic applications. Potentially, dysregulated RAS/MAPK and NO signaling in diseased cells can be normalized pharmacologically, either by reactivating muted responses or by taming excessive signaling in response to flow. Such interventions may prevent the manifestation of VaMs despite the presence of sensitizing mutations and short-circuit VaMs by intervening in downstream events.

## Future directions

The task in front of us is challenging and requires that we characterize how different flow patterns impact both normal and mutant vascular cells. Basic research in this direction will help answer several fundamental questions: How do RAS/MAPK and NO pathways maintain vascular homeostasis and integrity in response to distinct hemodynamic forces? How do different flow patterns impact mutated vascular cells? Can these mechanisms be exploited for translation into therapies?

The central concept for these critical questions has resulted in formation of the Recalibrating Mechanotransduction in Vascular Malformations by Pharmacological Intervention (ReVAMP) consortium, funded by the Leducq Foundation Transatlantic Network of Excellence program. Its goal is to uncover protective molecular cascades activated by hemodynamic forces that are altered in RAS/MAPK– and NO pathway–mutated cells (Figure 1). Comprehensive interrogation of these forces and models will potentially benefit development of curative approaches to normalize biomechanical homeostasis in mutant vascular cells. What is needed are proof-of-principle studies in animal and organoid models in which VaMs are treated by pharmacological intervention with the aim of enabling affected vessel beds to recalibrate to a healthy morphology. Ultimately, such experiments will help to elucidate how RAS/MAPK vasculopathies affect the vascular cellular machinery involved in sensing and transmitting mechanical forces.

## Version 1 04/15/2022

Electronic publication
